# Non-Destructive Monitoring of External Quality of Date Palm Fruit (*Phoenix dactylifera* L.) During Frozen Storage Using Digital Camera and Flatbed Scanner

**DOI:** 10.3390/s24237560

**Published:** 2024-11-27

**Authors:** Younes Noutfia, Ewa Ropelewska, Zbigniew Jóźwiak, Krzysztof Rutkowski

**Affiliations:** Fruit and Vegetable Storage and Processing Department, The National Institute of Horticultural Research, Konstytucji 3 Maja 1/3, 96-100 Skierniewice, Poland

**Keywords:** freezing, ‘Mejhoul’, date cultivar, image processing, machine learning, prediction, classification

## Abstract

The emergence of new technologies focusing on “computer vision” has contributed significantly to the assessment of fruit quality. In this study, an innovative approach based on image analysis was used to assess the external quality of fresh and frozen ‘Mejhoul’ and ‘Boufeggous’ date palm cultivars stored for 6 months at −10 °C and −18 °C. Their quality was evaluated, in a non-destructive manner, based on texture features extracted from images acquired using a digital camera and flatbed scanner. The whole process of image processing was carried out using MATLAB R2024a and Q-MAZDA 23.10 software. Then, extracted features were used as inputs for pre-established algorithms–groups within WEKA 3.9 software to classify frozen date fruit samples after 0, 2, 4, and 6 months of storage. Among 599 features, only 5 to 36 attributes were selected as powerful predictors to build desired classification models based on the “Functions-Logistic” classifier. The general architecture exhibited clear differences in classification accuracy depending mainly on the frozen storage period and imaging device. Accordingly, confusion matrices showed high classification accuracy (CA), which could reach 0.84 at M0 for both cultivars at the two frozen storage temperatures. This CA indicated a remarkable decrease at M2 and M4 before re-increasing by M6, confirming slight changes in external quality before the end of storage. Moreover, the developed models on the basis of flatbed scanner use allowed us to obtain a high correctness rate that could attain 97.7% in comparison to the digital camera, which did not exceed 85.5%. In perspectives, physicochemical attributes can be added to developed models to establish correlation with image features and predict the behavior of date fruit under storage.

## 1. Introduction 

Naturally, fresh fruit and vegetables are perishable and subject to postharvest quality deterioration caused by physiological disorders, microbial attacks, and handling operations [1]. In fact, the higher moisture content, respiration rate, and moderate–high amount of sugars in fresh crops are considered favorable factors for subsequent damages [2]. In the perspective of reducing postharvest losses, various technologies and chemical-based treatments are widely used to preserve the quality of fruit and vegetables [3]. Presently, techniques based on controlling “temperature”, “relative humidity”, and “gas composition” has allowed the period of storage to be extended, ensuring the availability of products throughout the year [4]. Chilling associated with relative humidity regulation, conventional and cryogenic freezing, controlled and modified atmosphere packaging, and oligosaccharide coating, in addition to different chemical treatments, are frequently applied in the storage value chain of fruit [3,5].

Date palm fruit (*Phoenix dactylifera* L.) are among the perishable fruit subject to a high level of postharvest deterioration and quality losses if storage conditions are not well mastered and applied [6]. Thus, physical defects such as skin separation, sugar spotting, and non-enzymatic browning, in addition to insect infestation, weight loss, and biochemical alterations, are largely associated with quality deterioration under storage [6,7]. Therefore, the most common preservative techniques employed to maintain date fruit quality and reduce the occurrence of the aforementioned defects are focused on the application of low-temperature treatments such as refrigeration and freezing [8,9]. Besides these thermal processes, other methods such as fumigation, modified atmosphere packaging, edible coating, and, in some cases, heat treatment have demonstrated their effectiveness, especially for dry date fruit [9]. To evaluate storage efficiency based on the selected preservative technique, microbiological, chemical, and physical compounds can be used as predictors of the shelf life and storage ability of different date cultivars. Nonetheless, the determination of such compounds is complex and time-consuming, and it requires qualified human resources and tedious analyses at a laboratory scale. Hence, emerging non-destructive imaging techniques constitute powerful alternatives in monitoring and predicting date fruit quality during postharvest handling and storage [10,11,12]. Based on their potential use, each imaging technique necessitates specific devices for image acquisition and has its own tools/software for image processing and feature classification [13]. For date palm storage and shelf life determination using machine learning approaches, the available studies remain insufficient and deal with limited applications of artificial intelligence (AI) models, requiring initial and complex programming steps. Moreover, these few studies are focused only on the effect of positive cold storage and modified atmosphere conditions on the quality of date fruit cultivars cultivated in the Gulf region. Their quality was evaluated by correlating electrical properties and a spectral profile with biochemical properties using computer vision technologies and AI-based algorithms and modules [14,15,16].

Compared to these studies, this investigation seeks to assess the behavior of two date palm cultivars under freezing conditions using non-destructive analysis based on an imaging profile acquired by two devices, a Canon digital camera (Canon EOS 2000D, Ōta, Tokyo, Japan) and Epson Perfection flatbed scanner (Epson, Suwa, Nagano, Japan), analyzed by ML and AI-based software (MATLAB R2024a and WEKA 3.9).

## 2. Materials and Methods

### 2.1. Plant Material 

‘Mejhoul’ and ‘Boufeggous’ date fruit cultivars were harvested at an advanced stage of maturity in the modern orchard at Erfoud (Southeastern Morocco), packed in carton boxes, transferred to the HortiFood Processing Centre (Skierniewice-Poland), and stored at 2–4 °C until freezing assays.

### 2.2. Freezing Assay 

For each cultivar, date samples of proximate quality (Table 1) were randomly divided into groups of 30 fruits without defects and packed using plastic bags.

**Figure 1:**
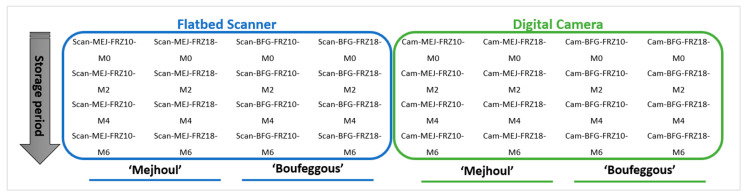
Experimental design for frozen date fruit under storage. MEJ: ‘Mejhoul’; BFG: ‘Boufeggous’; FRZ10: freezing at −10 °C; M0: month 0; Cam: camera; Scan: scanner.

This experimental design resulted in the following 32 treatments (Figure 1).

Packed date fruit from all the above-mentioned treatments were freeze-stored for 6 months on shelves in two MEDGREE MARECOS freezers (MLF 66 S-ATEX Model, MEDGREE MARECOS, Tremês, Portugal) at −10 °C and −18 °C. The freezer consisted of a freezer compartment with 3 removable shelves, a cooling system with static cold on the walls using R 600a Gaz, a control panel, and connected probes to permanently measure the indoor temperature. Frozen date fruit samples were assessed at a frequency of 0, 2, 4, and 6 months for image texture features.

### 2.3. Image-Texture Feature Determination 

**Figure 2:**
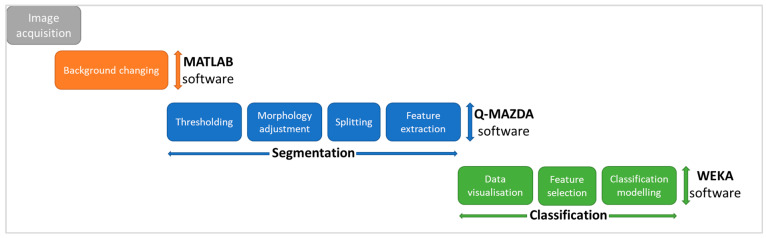
Logical flowchart for date fruit image processing and analysis.

The workflow for image processing/analysis is summarized in Figure 2.

#### 2.3.1. Images Acquisition

Images of date fruit were acquired using a Canon digital camera (Canon EOS 2000D, Ōta, Tokyo, Japan) with a Canon Zoom Lens EF-S 18–55 mm/Ø58 mm and Epson Perfection flatbed scanner (Epson, Suwa, Nagano, Japan). The camera settings were as follows:

- ISO speed: ISO-800.
- Exposure time: 1/125 s.
- Focal length: 23 mm.

#### 2.3.2. Changing Background

For better image segmentation, the MATLAB R2024a software package (MathWorks, Inc., Natick, MA, USA) was used to change the white background to black using a specific code. The obtained images were saved in BMP format.

#### 2.3.3. Segmentation

**Figure 3:**
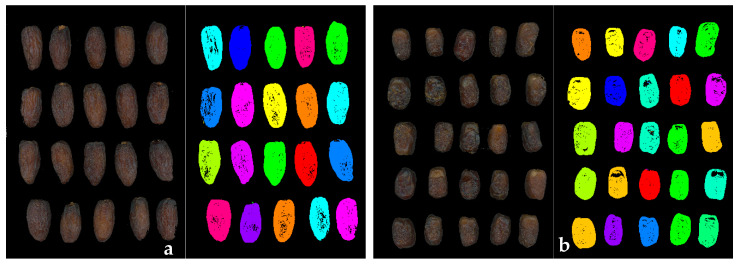
Illustration of background images and their respective ROIs for ‘Mejhoul’ (**a**) and ‘Boufeggous’ (**b**) cultivars.

Images with a black background were uploaded to the interface of Q-MAZDA 23.10 software [17] (Łódź University of Technology, Institute of Electronics, Łódź, Poland) to perform image segmentation. Initially, Regions Of Interest (ROIs) were obtained in a Q-MAZDA module automatically by applying predefined image segmentation algorithms practically consisting of three steps: thresholding, morphology adjustment, and splitting. An example of obtained ROIs for the ‘Mejhoul’ and ‘Boufeggous’ cultivars is given in Figure 3.

Subsequently, image attributes were extracted based on color, texture, and morphological features. In total, 599 features were extracted based on the image histogram, gradient, co-occurrence matrix, run-length matrix, autoregressive model [18], and Gabor and Haar wavelet transforms [19].

#### 2.3.4. Classification 

Before classification, color, texture, and morphological features were used as inputs for WEKA software (WEKA 3.9 machine learning software, University of Waikato, Hamilton, New Zealand) to select highly discriminative features based on the “BestFirst” algorithm. Selected features were employed to build classification models according to the “Functions-Logistic” classifier found to be the most accurate compared to other classifiers belonging to the algorithm groups of “Lazy”, “Meta”, “Rules”, and “Trees” (Table 2).

## 3. Results and Discussion 

### 3.1. Comparative Assessment of Performance Metrics Under Freezing Conditions 

A comparison of the performance metrics obtained for frozen ‘Mejhoul’ and ‘Boufeggous’ cultivars under two storage temperatures is given in Table 3 according to the used imaging device.

Based on the results reported in Table 3, the highest “True Positive Rate (TPR)” values were obtained for the ‘Boufeggous’ cultivar stored at −10 °C and −18 °C using a flatbed scanner, with respective values of 0.838 and 0.814. The lowest values for TPR were found for the ‘Mejhoul’ cultivar using the digital camera. For the same imaging device, the “precision” as well as the “TPR” were higher for ‘Boufeggous’ compared to ‘Mejhoul’. The performance metrics shown above are slightly lower than in previous investigations focused on discrimination between five Moroccan date palm cultivars [20] and the classification of fresh and lacto-fermented red bell pepper (*Capsicum annuum* L.) [21]. However, our findings were comparable to global performance metrics, especially for “precision” and “recall”, as reported in a holistic study dealing with fruit classification using attention-based MobileNetV2 [22].

**Figure 4:**
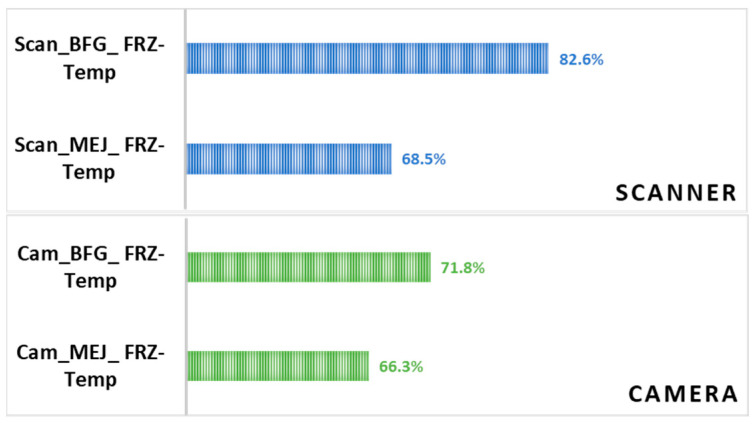
Accuracy rates obtained using the flatbed scanner and digital camera for discrimination between frozen date samples under different temperatures.

For storage temperature, freezing at −10 °C exhibited a slightly higher prediction level compared to freezing at −18 °C. This trend was observed for both cultivars regardless of the image-capturing device. Overall, higher accuracy rates were obtained by the use of the flatbed scanner as an instrument of image acquisition, by using ‘Boufeggous’ as the date cultivar, and for −10 °C as a storage temperature. By contrast, lower prediction levels were recorded when using the digital camera, the ‘Mejhoul’ cultivar, and a temperature of −18 °C (Table 3). To illustrate the case of the imaging device, the accuracy levels obtained by using the flatbed scanner and digital camera for discrimination between the two storage temperatures at −10 and −18 °C is given in Figure 4. Thus, a respective accuracy rate of 82.6% and 71.8% was recorded for ‘Boufeggous’ images acquired using the scanner and camera. For ‘Mejhoul’, this accuracy was, respectively, 68.5% and 66.3%.

In the field of computer vision, it is evident that the image acquisition device, image quality/resolution, and imaging method impact image segmentation, feature selection, and the classification process, resulting in different accuracy levels [23]. Therefore, it is highly recommended to employ good-quality image acquisition systems equipped with high-resolution cameras [24] with the aim of ensuring the proper visibility of relevant features and generating a large database that can enable accurate classification and deep prediction. Furthermore, the fine detection of crop and weed plants by precision agricultural robots has been outlined as promising since they can precisely discriminate between specimens based on color, size, and morphology [25]. In comparison with our findings, the use of a flatbed scanner allowed for the production of images with high resolution, with an average size of 203 megabits. However, the digital camera images were of an average size of 69 megabits. Consequently, these differences in image resolution directly affected the levels of accuracy, as is revealed in Figure 4. These results are in agreement with a previous study, confirming that the image resolution, the size of the data, and the quality of the image affects classification accuracy. Images with low resolution contain a huge amount of noise, resulting in reduction in the performance of image classification [26].

### 3.2. Image Feature Selection for Frozen Date Fruit During Storage

Before classification, the selection of accurate features from captured images is a key step for discrimination between selected classes. In Table 4, an overview of feature selection is given for the case of building models aiming to elucidate the potential effect of the storage period on each cultivar and according to each of the imaging devices.

From 599 morphological (shape) and color–texture features obtained after image segmentation and feature computation, only 5 to 36 accurate features were retained (Table 4). Furthermore, color–texture descriptors were highly representative in the selected features in comparison with morphological ones. In addition, color–texture features were ranked as the five best discriminative attributes for all classes reported in Table 4 besides the last class—“Cam_MEJ_FRZ-18 [M0-M2-M4-M6]”. Among the color–texture attributes, the Y component, which refers to “brightness”, was mostly used as a targeted monochrome color component to convert both digital camera and flatbed scanner images, before applying extraction algorithms. In a similar study, it was reported that color–textural features were powerful predictors compared to shape features for defect and grading discrimination between the quality of tomatoes [27].

Based on the same image acquisition device, the required number of features to build predictive models is higher for the ‘Boufeggous’ cultivar than for the ‘Mejhoul’ cultivar. For the example of “Scan_BFG_FRZ-18 [M0-M2-M4-M6]”, 32 features were used in comparison to only 10 features for “Scan_MEJ_FRZ-18 [M0-M2-M4-M6]”. Similarly for the digital camera, it was found that 31 features were used for the case of “Cam_BFG_FRZ-18 [M0-M2-M4-M6]”, compared to 5 features for “Cam_MEJ_FRZ-18 [M0-M2-M4-M6]”. Also, and according to Table 4, building models using a digital camera necessitated, in total, 101 selected features, while 87 features were employed for the scanner. Thus, it can be concluded that discrimination between classes with the ‘Boufeggous’ cultivar required more features than classes with the ‘Mejhoul’ cultivar. Likewise, building models based on “digital camera” images required more features than for those based on the “flatbed scanner”.

### 3.3. Classification Accuracy of Frozen ‘Mejhoul’ and ‘Boufeggous’ Cultivars During Storage 

**Figure 5:**
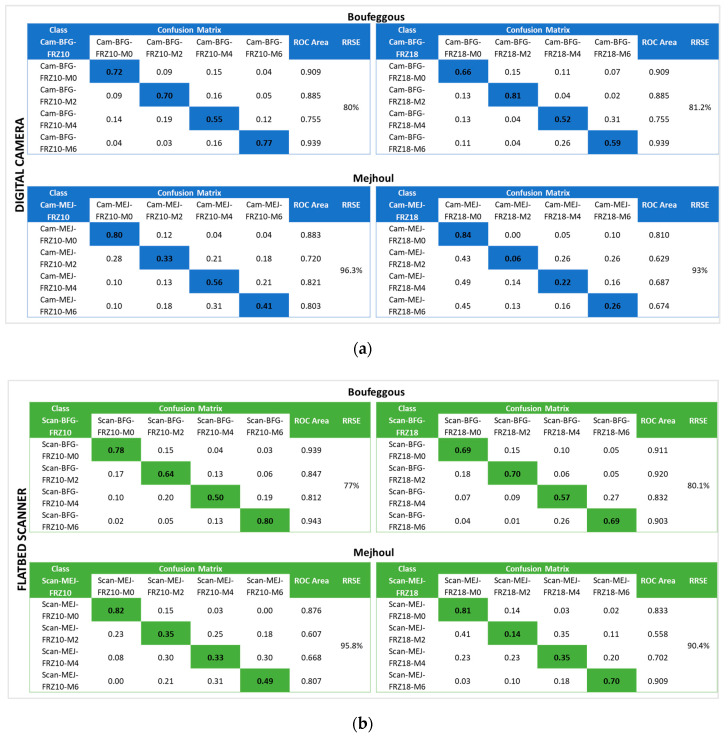
Confusion matrix, ROC area, and RRSE of freeze-stored ‘Mejhoul’ and ‘Boufeggous’ cultivars obtained using a flatbed scanner (**a**) and digital camera (**b**).

Discrimination between ‘Mejhoul’ and ‘Boufeggous’ cultivars after various periods of storage at −10 °C and −18 °C is presented in Figure 5 based on digital camera and flatbed scanner images. Comparable classification accuracies, ranging between 0.33 and 0.84, were observed for the use of the two imaging devices.

In most of the cases, the highest accuracy levels were found for both cultivars at the beginning of storage (M0), while the lowest levels were noted for the second (M2) and the fourth (M4) months of frozen storage. Thus, an average accuracy level of 76.5% was calculated at M0 for all cultivars compared to 46.6% and 45% for M2 and M4, respectively. Generally, the accuracy rate indicated a decrease during M2 and M4 for all treatments mentioned in Figure 5 before a significant increase at the end of storage (M6), especially for cultivar images acquired with the flatbed scanner.

This trend can be visualized clearly in the obtained values for the ROC area. For the case of “Scan-BFG-FRZ10”, this parameter had an initial value of 0.939 that decreased, respectively, to 0.847 and 0.812 after 2 and 4 months of storage, before increasing at 0.943 at the end of freezing (M6). By referring to the confusion matrix given in Figure 5, date fruit samples at M2 of storage were more often confused with samples of M0 than date samples of M4 and M6. This confusion classification was more pronounced for the case of the ‘Mejhoul’ cultivar. Thus, the class “Cam-MEJ-FRZ-10” showed an accuracy rate of 0.33 at M2 and 0.28 at M0, while this rate was 0.21 and 0.18 for M4 and M6, respectively.

The above-mentioned observations can be explained by low changes in external properties of both the ‘Mejhoul’ and ‘Boufeggous’ cultivars, which might have occurred between the 2nd and the 4th month of freezing. These changes seem to have reduced the prediction rate between the same class, and the developed algorithms did not precisely recognize the accurate class in this storage period. By the end of storage (M6), the level of external changes seemed to be higher, thus allowing for high distinguishing capabilities for frozen date fruit cultivars. Overall, both the relative high accuracy obtained for some classes (e.g., Cam-BFG-FRZ18-M0) and the high relative root square error attained (ranging from 77 to 96.3%) may reflect the powerfulness of the developed models using the selected algorithm.

### 3.4. Classification Correctness Between Freeze-Stored Date Fruit

**Figure 6:**
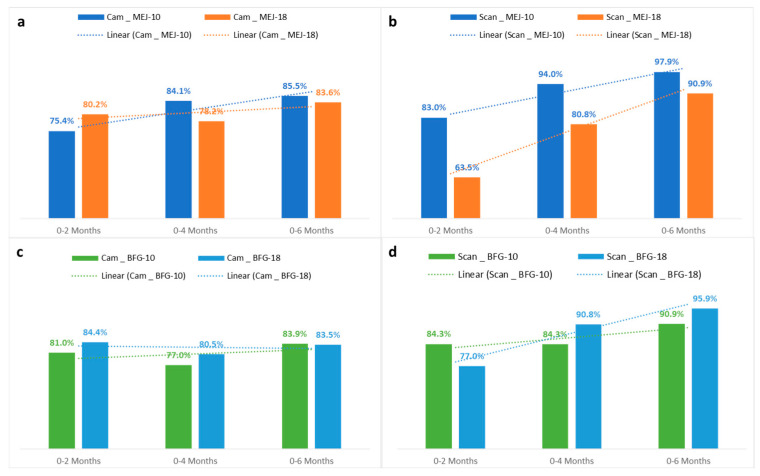
Classification correctness between fresh and freeze-stored date fruit at 2, 4, and 6 months at −10 °C and −18 °C for ‘Mejhoul’ samples acquired with (**a**) a digital camera and (**b**) flatbed scanner and ‘Boufeggous’ samples acquired with (**c**) a digital camera and (**d**) flatbed scanner.

To better elucidate the effect of frozen storage on the two assessed date cultivars’ behavior, classification correctness between fresh date fruit samples and those stored after 2, 4 and 6 months is given in Figure 6 based on freezing temperature and the imaging device used.

The general overview given in Figure 6 clearly shows differences in classification accuracy, mainly depending on the freeze-storage period and imaging device used for image acquisition. However, the effects of cultivar and storage temperature were found to be less important/discriminative in affecting the behavior of date palm samples under storage.

The developed models on the basis of flatbed scanner use allowed us to obtain a high correctness rate that could reach 97.7%, in comparison to the digital camera, which did not exceed 85.5% in terms of accurate classification. For example, discrimination between frozen ‘Boufeggous’ (stored at −18 °C) at 0 and 4 months showed that the rate of correct classification was, respectively, 90.8% and 80.5% for fruit samples captured using the flatbed scanner and digital camera.

If the analysis is based on the comparison between fresh and frozen date fruit samples, the trendlines reported in Figure 6 exhibit a general progressive increase in prediction level while progressing in the storage period. Higher classification accuracy (CA) was recorded for discrimination between fresh date fruit and those stored for 6 months, and lower CA was indicated for the classification of date fruit between 0 and 2 months. Respective CA values of 75.4% and 81% were found for discrimination between “Cam-Mejhoul” and “Cam-Boufeggous” stored at −10 °C between 0 and 2 months. After 6 months of storage at the same temperature, the CA values increased, respectively, to 85.5% and 83.9% for “Cam-Mejhoul” and “Cam-Boufeggous”. This increase in CA was more notable when the flatbed scanner was used. Thus, prediction levels increased when discrimination was carried out, respectively, between the class of 0–2 months and 0–6 months. For ‘Mejhoul’ stored at −18 °C, the prediction rate increased from 63.5% for the class 0–2 months to 90.9% for the class 0–6 months. The same trend was visualized for ‘Boufeggous’, since the percentage of correctly classified instances increased from 77% to 95.9%. This increase automatically reflected a decrease in the misclassification rate while advancing in the storage period.

The above-mentioned CA rates indicated that predictive models developed using “Functions-Logistic” algorithms were more accurate when they were applied for the comparison of date fruit samples stored fresh and after 6 months of storage. The same algorithms were less predictive when a comparison was established for fresh date samples and dates stored for 2 months. These differences may be directly linked to potential external changes that can occur on the surface of analyzed cultivars during storage. Indeed, low CA (obtained between 0 and 2 months of storage) indicated small changes in the outer surface of date fruit as captured by imaging device and transformed to image features with close numerical values that could not be used to build strong predictive models. Between 0 and 6 months, freeze storage seems to have a more detectible effect on the external quality of both date cultivars, making the CA higher than that observed between 0 and 2 months.

In a similar study, a non-destructive method based on hyperspectral imaging and supervised classification algorithms was effective for the earliest detection of stored eggplants with chilling injury from the 2nd day of storage at 2 °C [28]. In another investigation, the effect of storage temperature and period were evaluated for red currants (*Ribes rubrum* L.) using data generated from images and analyzed with several machine learning algorithms. The results showed a high distinguishing rate for stored fruit between 1 and 2 weeks [29]. To our knowledge, no specific study was dedicated to assess the effect of freezing during the storage period according to an image analysis of date fruit samples.

Available results from the literature confirm our findings but on the basis of destructive analyses such as that for physical and chemical compounds [30,31,32].

## 4. Conclusions

Overall, this investigation demonstrated that higher accuracy rates were obtained by the use of a flatbed scanner as an instrument of image acquisition, for ‘Boufeggous’ as a date cultivar, and for −10 °C as a storage temperature. This reflected that the main changes in external quality occurred for ‘Boufeggous’ compared to ‘Mejhoul’ and that these changes on the outer surface were more pronounced at −10 °C in comparison to −18 °C. The obtained results can be of practical importance for production, processing, and marketing. It was proven that a flatbed scanner can be a suitable device to assess date fruit quality in a non-destructive manner based on image features. It can be suggested (due to its high resolution) for use as an accurate detector for date fruit sorting and grading, as well as for controlling quality under storage. In this perspective, physicochemical attributes can be added to the developed models to establish a correlation with image features and predict the behavior of date fruit under storage. Due to the development of prediction models, estimation of the physicochemical characteristics of date fruit can be possible based on image parameters.

## Figures and Tables

**Table 1:** Physicochemical and morphological characteristics of fresh date fruit cultivars.

	Length (mm)	Diameter (mm)	Flesh Thickness (mm)	Fruit Weight (g)	Moisture Content (%)	Water Activity	TSS (°Bx)
‘Mejhoul’	45.1	21.8	3.77	18.2	18.4	0.586	77.7
‘Boufeggous’	33.2	20.9	1.38	12.5	24.5	0.672	71.7

**Table 2:** Classification accuracy obtained for different algorithms used in the case of discrimination between storage temperature (for both cultivars based on camera images).

Classifier	Classification Accuracy	
	‘Mejhoul’	‘Boufeggous’
Functions-Logistic	66.4%	71.8%
Lazy.IBk	57.8%	69.4%
lazy.KStar	55.7%	65.7%
meta.LogitBoost	62.6%	70.3%
trees.RandomTree	59.5%	63.3%
rules.ZeroR	51.2%	49.9%

**Table 3:** Performance metrics obtained for distinguishing frozen ‘Mejhoul’ and ‘Boufeggous’ cultivars at −10 °C and −18 °C, based on digital camera and flatbed scanner images.

Class	TP Rate	Precision	Recall	F-Measure	MCC	ROC Area	PRC Area	RRSE
Scan-BFG-FRZ10	0.838	0.819	0.838	0.828	0.652	0.917	0.916	69.2%
Scan-BFG-FRZ18	0.814	0.833	0.814	0.823	0.652	0.917	0.922	
Cam-BFG-FRZ10	0.729	0.713	0.729	0.721	0.436	0.788	0.782	87.1%
Cam-BFG-FRZ18	0.707	0.723	0.707	0.715	0.436	0.788	0.773	
Scan-MEJ-FRZ10	0.693	0.685	0.693	0.689	0.369	0.762	0.748	89.8%
Scan-MEJ-FRZ18	0.676	0.684	0.676	0.680	0.369	0.762	0.767	
Cam-MEJ-FRZ10	0.657	0.676	0.657	0.667	0.327	0.719	0.703	92.8%
Cam-MEJ-FRZ18	0.670	0.651	0.670	0.660	0.327	0.719	0.715	

TP: true positive; MCC: Matthews correlation coefficient; ROC Area: receiver operating characteristic area; PRC Area: precision–recall area; RRSE: root relative square error.

**Table 4:** Feature selection summary.

	Total Number of Selected Features	Color–Texture Feature Number	Morphological Feature Number	Best First 5 Accurate Features
Scan_BFG_FRZ-10 [M0-M2-M4-M6]	26	22	4	YD8ArmTeta1 YD5GlcmH1DifVarnc YLbpOc4n2 YLbpOc4n5 YLbpOc4n11
Scan_BFG_FRZ-18 [M0-M2-M4-M6]	32	27	5	YD8HistKurtosis YD8HistPerc99 YD8ArmTeta1 YD8ArmTeta2 YD5GlcmZ3DifVarnc
Scan_MEJ_FRZ-10 [M0-M2-M4-M6]	19	16	3	YD8Gab4H2Mag YD8DwtHaarS4LH YLbpOc4n14 BD8HistVariance BD8HistKurtosis
Scan_MEJ_FRZ-18 [M0-M2-M4-M6]	10	7	3	YD8Gab4V2Mag BD8HistMaxm10 SD8HistSkewness uD8HistVariance qD8HistVariance
Cam_BFG_FRZ-10 [M0-M2-M4-M6]	36	33	3	YD8HistKurtosis YD8GradSkewness YD8Gab8V4Mag YD8DwtHaarS2HH YD5GlcmH2ClustShd
Cam_BFG_FRZ-18 [M0-M2-M4-M6]	31	26	5	YD8GradSkewness YD8Gab16V8Mag YD5GlcmV1ClustShd GD8HistKurtosis GD8HistDomn01
Cam_MEJ_FRZ-10 [M0-M2-M4-M6]	29	21	8	YD8HistArea YD8ArmTeta4 YD8GradSkewness YD8Gab6V3Mag YD8Gab8Z4Mag
Cam_MEJ_FRZ-18 [M0-M2-M4-M6]	5	3	2	iD8HistKurtosis qD8HistMean hD8HistMean MorItkCentroidY MorMzW8

## Data Availability

The original data presented in the study are included in the article. Further inquiries can be directed to the corresponding author. The data are not publicly available due to the continuation of the project to which the data relate.
